# Exploring the Effects of Major Depressive Disorder on Daily Occupations and the Impact of Psychotherapy: A Literature Review

**DOI:** 10.7759/cureus.55831

**Published:** 2024-03-09

**Authors:** Kalliopi Iliou, Dimitrios Balaris, Anna M Dokali, Vasileios Fotopoulos, Athanasios Kouletsos, Aikaterini Katsiana

**Affiliations:** 1 Occupational Therapy, University of Western Macedonia, Kozani, GRC; 2 Anatomy, University of Ioannina, Ioannina, GRC; 3 Psychological Science, Western Sydney University, Sydney, AUS; 4 Medical Veterinary Life Science, University of Glasgow, Glasgow, GBR

**Keywords:** effects of major depressive disorder, daily occupations, impact of psychotherapy, everyday activities, psychotherapy, cognitive deficit, occupational functionality, occupation, major depressive disorder (mdd)

## Abstract

Major depressive disorder (MDD) is a prevalent psychological mood disorder that can disrupt one's functioning and result in decreased engagement in daily activities. Psychotherapy, in different approaches, is a common approach for individuals experiencing MDD. Nevertheless, a literature review of the research supporting the effectiveness of psychotherapeutic interventions in patients with MDD-impacted areas of their daily occupations, such as back to work, cognitive deficits, and well-being, has not been conducted.

A literature review was carried out to evaluate the effectiveness of psychotherapy on daily occupations for individuals diagnosed with MDD. Due to variations in study design and outcome measures, a best evidence synthesis was carried out instead of a meta-analysis.

Forty-one identified articles were fully assessed in total. These studies were conducted in various countries so that a global approach could be considered comprehensive. The findings showed strong evidence supporting the effectiveness of psychotherapy on return-to-work interventions in improving depressive symptoms. There was limited evidence for the effectiveness of psychotherapy on lifestyle interventions in reducing anxiety and suicidal ideation, as well as limited evidence for enhancing work participation. Notably, there were no studies evaluating individualized client-centered psychotherapy interactions with occupations, revealing a research gap. Challenges such as incomplete reporting within studies and study heterogeneity prevented a meta-analysis.

While the overall evidence base for the effectiveness of psychotherapy for MDD in treating functionality is limited, the findings provide strong support for the efficacy of occupational therapy return-to-work interventions. This is particularly important given the economic costs associated with mental health issues and work-related absences. Further research is required to strengthen the existing evidence base.

## Introduction and background

The present study aims to examine and integrate current literature and research on psychotherapeutic intervention in patients with major depressive disorder (MDD) and its impact on occupations. In the field of psychotherapy, "occupations" encompass the daily tasks and activities that individuals engage in, whether independently, within families, or as part of their communities. These activities serve to fill one's time with meaning and purpose, and they encompass tasks that individuals are required to perform, desire to accomplish, and are anticipated to complete [1]. MDD, on the other hand, is a prevalent psychological mood disorder characterized by symptoms that can interfere with daily functioning and result in reduced involvement in everyday activities [2]. These symptoms include persistent feelings of sadness, dysphoria, diminished motivation, and a range of physical and cognitive impairments [3]. As per the World Health Organization, approximately 300 million individuals are impacted by MDD, making it the foremost contributor to disability on a global scale [4].

The annual prevalence of MDD varies significantly from one country to another but averages around 6% globally [5]. The lifetime risk of experiencing MDD is notably higher, at three times the rate (15-18%), indicating that MDD is a common condition, with nearly one in five individuals encountering at least one episode during their lifetime [6]. Therefore, in primary healthcare settings, roughly one in ten patients typically exhibit depressive symptoms, though the prevalence of MDD tends to be higher in secondary care environments [7]. Notably, the annual prevalence of MDD remains similar when comparing high-income countries (5.5%) to low-income and middle-income countries (5.9%) [8]. This suggests that MDD cannot be simply attributed to the lifestyle in developed nations or poverty [9,10]; instead, it involves a complex interplay of social and cultural factors, such as socioeconomic status, along with genomic and other underlying biological factors [11]. The typical age range for the initial occurrence of an MDD episode extends from adolescence in the middle years to the mid-40s [12]; However, it is worth noting that nearly 40% of individuals experience their first episode of MDD before reaching the age of 20. On average, the onset of MDD tends to happen during the mid-20s, with a median age of 25, though it can vary between 18 and 43 [13]. Throughout one's life, MDD is half as prevalent in men compared to women. In both genders, there is a notable increase in MDD prevalence during the second and third decades of life, followed by a smaller peak in the fifth and sixth decades [14]. The variation in MDD rates between men and women, often termed the "gender gap in MDD," seems to be associated with differences in susceptibility, encompassing biological and psychological factors, as well as environmental influences that impact individuals at both micro and macro levels [15].

The beginning of MDD typically occurs slowly, although there are instances when it can manifest suddenly, and the trajectory of MDD over one's lifetime can differ significantly [16]. In most cases, MDD follows an episodic pattern, with individuals experiencing periods of acute depressive symptoms interspersed with periods of feeling well [16]. The degree of disruption to one's functioning is contingent upon the seriousness of the MDD episode and can be significant, affecting engagement in everyday activities such as self-care, work, social interactions, and leisure pursuits [17]. Psychotherapeutic interventions for MDD aim to empower individuals to partake in the daily activities they desire or need, ultimately enhancing their health, well-being, and overall quality of life [1]. Presently, there is limited research evidence regarding the effectiveness and impact of psychotherapeutic interventions in occupations as a whole. A scoping review of the literature concerning the effectiveness of psychotherapy on functionality for individuals diagnosed with MDD also revealed a scarcity of published research in this area [18-20].

On a global scale, not all professionals working as therapists in the field of mental health dedicate their entire clinical practice to delivering occupational therapy services, which is partly due to an increase in generic or generalist roles [21-23]. While the standards for community mental health services in Great Britain emphasize the importance of occupational therapy, there have been cutbacks in staffing and cost-saving measures that have resulted in a reduction in occupational therapy positions [24]. Consequently, psychotherapists are allocated less time for delivering specific occupational therapy services and more time for general mental health practitioner duties, which are often considered higher priorities in services facing strain. In the United States, this shift has had detrimental effects on the quality of patient care, as well as on the professional identity and collaborative work of multi-disciplinary teams [22].

Having solid evidence to support best practices is not only essential for ensuring the delivery of effective and high-quality interventions, as emphasized by the World Health Organization in 2004, but it's also crucial for making the most of limited healthcare resources to achieve optimal outcomes for patients [4]. The limited evidence regarding the effectiveness of therapy in mental health places the profession and the individuals benefiting from its interventions at ongoing risk due to cost-saving measures resulting from economic austerity. The primary objective of this literature review was to address the question: Does psychotherapy enhance functioning and participation in everyday activities for adults diagnosed with major depressive disorder?

## Review

Methods

In December 2023, PubMed, Science Direct, Google Scholar, and Scopus were searched for literature publications focused on psychotherapy and its impact on occupations in humans suffering from MDD. The search strategy used the keywords: "major depressive disorder" OR "depression" OR "depressive illness" OR "affective disorder" OR "low mood" OR "mood disorder" AND "psychotherapy" OR "therapy" OR "occupation" OR "wellbeing" OR "leisure" OR "daily living" OR "adaptive function" OR "functioning" OR "productive" OR "participation" OR "independence" OR "effectiveness" OR "everyday life". Four independent reviewers performed the manual search of references and relevant scientific articles for included studies. Search results from the aforementioned databases were imported to Mendeley Reference Manager [25] to remove any duplicates. Four independent reviewers performed title and abstract screening, if that was available, followed by the full-text screening of the scientific articles identifying correlations or interactions between psychotherapy and occupations of individuals with MDD. Scientific information from abstract-only articles, theses, posters, editorials, letters, conference papers without original data, and commentaries were excluded. There was no restriction on the publication year of the published scientific work. A total of 41 identified studies were included in this literature review. The included articles were published in English. This literature review provides a summary of the evidence-based impacts of psychotherapy on the occupations of patients with MDD based on the available information.

The impact of MDD on occupations

Individuals diagnosed with MDD indicate significant impairments in their occupations, such as work functioning, social interactions, self-care activities, household responsibilities, recreational activities, academic performance, and various others [26,27]. Over 90% of patients report experiencing functional limitations in at least one domain during a major depressive episode, and these limitations may endure even after there is an improvement in depressive symptoms, resulting in a higher likelihood of relapse and escalating healthcare expenses [28-31]. Certain research has even noted that the extent of occupational limitations linked to MDD is similar to or may even surpass those linked to other significant medical conditions [32]. Furthermore, the severity of symptoms in MDD appears to account for a limited percentage of the variability in functional impairment [33].

Deficiencies in cognitive performance and the incapacity to function in everyday life situations are observed, to varying extents, in numerous severe mental disorders, including major depressive disorder [34,35]. Functional and cognitive approaches can be situated at two separate levels of explanation; however, the functional approach focuses on explanations of behavior in terms of its dynamic interaction with the environment, and the cognitive approach aims to explain environment-behavior relations in terms of mental mechanisms [36]. Although mood symptoms can affect cognitive performance, both mood symptoms and cognitive deficits exert a substantial impact, partially independent of the psychosocial functioning of psychiatric patients. Enhancing the management of mood symptoms could serve as a crucial approach to achieving better functional outcomes [37]. Moreover, cognitive impairment emerges as an independent dimension in numerous psychiatric disorders, emphasizing the importance of considering these symptoms as potential targets for treatments aimed at mitigating functional deficits [35].

Improvements in daily occupations, which may include enhanced work productivity, improved social engagement and interactions, better self-care routines, increased participation in household responsibilities, more enjoyable engagement in recreational activities, enhanced academic performance, and overall improved quality of life, are notably observed during remission, particularly when specific criteria for remission are met, including the complete absence of symptoms, the absence of residual symptoms, sustained maintenance of remission, the quality of remission, and early achievement of remission [27,33]. Nevertheless, numerous studies have demonstrated that factors beyond the mere remission of a depressive episode can directly impact functioning, including the combination of pharmacotherapy with psychotherapy [38,39]. The achievement of remission or a reduction in symptom severity in MDD may not suffice to indicate genuine recovery [40]. There should be an increased focus on functioning as a vital measure of treatment success. In evaluating treatments, such as psychotherapy, therapists should consider the effectiveness of assisting patients with their daily occupational activities [41].

Evaluating functional abilities of individuals with MDD

While there is available evidence and perspectives on the variation in functional disability patterns based on the severity of depression [42], researchers are increasingly presenting more compelling contradictory findings. These findings suggest that functional disability and depressive symptoms may not always be interlinked, indicating that mood symptoms alone may not completely account for the extent of functional impairment [35]. Additionally, improvements in functionality may not consistently align with enhancements in depressive symptoms [43]. Given this context, it becomes crucial to assess adaptive functioning as an integral part of treating depression to enhance the effectiveness of interventions. From the patient's viewpoint, achieving mood improvement is essential for a successful return to their pre-illness lifestyle, especially for individuals in the young, active, and productive age group.

The increasing emphasis on functional recovery as a target in clinical treatment has led scientists and clinicians to advocate for the accurate measurement and quantification of adaptive functioning. Surprisingly, less than 5% of clinical trial research for MDD has employed scales to assess functional levels [31]. There is a significant lack of research revolving around the functional aspects of MDD, with previous studies predominantly examining general functional outcomes rather than specific functional dimensions.

A study aimed at comprehensively evaluating the functional abilities of individuals with MDD, using a standardized adaptive functioning scale, had the objective of illustrating diverse aspects and degrees of impaired functioning in MDD patients. The researchers hypothesized that patients with MDD experience broad functional deficits affecting various facets of their adaptive functioning. By reproducing established findings, they indicated dysfunction across a range of functional domains in individuals with MDD, emphasizing the significance of systematically evaluating functional outcomes and obtaining input about functioning from sources other than the patients themselves [44].

Psychotherapy and MDD

The American Psychiatric Association has defined psychotherapy as the purposeful and informed application of clinical methods and interpersonal approaches grounded in established psychological principles. The aim is to assist individuals in modifying their behaviors, cognitions, emotions, and/or other personal characteristics in ways that are considered desirable by the participants [45].

It is widely acknowledged that MDD stands as one of the predominant psychiatric conditions globally, contributing to work-related disability, diminished work productivity [46], and disruptions in functioning that result in decreased participation in daily activities [19]. However, it is important to highlight that there is currently limited investigation into the impact of psychotherapy on occupational improvements in individuals with MDD.

Previous research on treating depression has predominantly focused on clinical outcomes related to symptoms, with a specific emphasis on improving work functioning for individuals with MDD. Psychotherapy has been shown to enhance functioning and quality of life for adults diagnosed with MDD. Although there are limited comparative trials for certain analyses, a study conducted in 2016 by Kamenov et al. indicated that combined treatment is more effective. They examined various classes of antidepressant medications commonly prescribed for MDD, including selective serotonin reuptake inhibitors (SSRIs), serotonin-norepinephrine reuptake inhibitors (SNRIs), tricyclic antidepressants (TCAs), monoamine oxidase inhibitors (MAOIs), and atypical antidepressants. Overall, Kamenov et al. (2016) reviewed the efficacy, safety, and tolerability of these various pharmacotherapy options for MDD, considering factors such as response rates, remission rates, side effects, and potential drug interactions [47]. Nevertheless, both psychotherapy and pharmacotherapy alone have demonstrated efficacy in enhancing functioning and quality of life. The generally modest effects observed suggested that future customization of therapies may be necessary to more effectively address the specific needs of individuals with MDD experiencing problems related to functioning and quality of life.

A publication focused on mental wellbeing in the workplace highlighted that psychotherapy significantly enhances occupational functioning by addressing work-related stresses and psychological effects such as depression [48]. Additionally, comprehensive research and meta-analyses consistently show that psychotherapy, whether used independently or in combination with medication, effectively improves depressive symptoms, overall functioning, social interactions, work productivity, and engagement in daily activities [47]. This aligns with earlier meta-analytic findings on depressive symptoms, indicating no clear advantage for either intervention type [49].

Nevertheless, recent meta-analyses highlight strong patient preference for psychological treatment over medication [50]. In addition, evidence indicates that many individuals who prefer psychological therapy opt not to receive any treatment rather than take medication [51].

Research suggests that various forms of psychotherapy can be effective in enhancing occupational functioning among individuals with MDD. For example, a study by Cuijpers et al. (2013) found that cognitive-behavioral therapy (CBT) was associated with improvements in work functioning and productivity among individuals with depression [49]. Another study demonstrated that mindfulness-based cognitive therapy (MBCT) led to significant improvements in occupational functioning and job satisfaction compared to treatment as usual [52].

Furthermore, interpersonal psychotherapy (IPT) has shown promise in improving social and occupational functioning in individuals with MDD. A meta-analysis in 2016 indicated that IPT was effective in reducing depressive symptoms and improving social and occupational functioning [53].

It's important to note that the choice of psychotherapy may depend on individual preferences, treatment goals, and the specific needs of the patient. Therefore, a comprehensive assessment by a mental health professional is crucial in determining the most suitable form of psychotherapy for enhancing occupational functioning in individuals with MDD.

Discussion

The present study review, in the preceding pages, has consolidated a substantial body of literature indicating the importance of considering both mood symptoms and cognitive deficits in understanding and treating the condition that major depressive disorder (MDD) has on occupational functioning. Hence, this literature review offers insights into the relationship between major depressive disorder (MDD) and daily occupations, as well as the effectiveness of psychotherapy in mitigating these effects.

The literature review highlights the profound impact of MDD on various aspects of daily life, including work functioning, social interactions, self-care activities, household responsibilities, recreational activities, academic performance, and various others. By elucidating these impairments, the study underscores the importance of addressing occupational functioning in the treatment and management of MDD. Moreover, the study examines the role of psychotherapy interventions in ameliorating the negative effects of MDD on daily occupations. Psychotherapeutic approaches such as CBT, IPT, and mindfulness-based interventions have shown promise in enhancing work productivity, interpersonal relationships, self-care practices, and overall quality of life for individuals with MDD. The findings of the literature review have practical implications for mental health professionals involved in the treatment of MDD. By recognizing the specific occupational impairments associated with MDD, clinicians can tailor psychotherapeutic interventions to target these areas of dysfunction and promote functional recovery. Integrating occupational therapy into the treatment plan may also be beneficial in addressing vocational and daily living challenges. Furthermore, the present review underscores the importance of early detection and intervention in individuals with MDD to prevent or minimize occupational impairments. By addressing depressive symptoms and associated functional difficulties in the early stages of the disorder, mental health professionals can help individuals maintain or regain their ability to engage in meaningful daily activities and roles.

The extent of occupational limitations in MDD may rival or exceed those associated with other medical conditions, and the severity of symptoms explains only a limited percentage of functional impairment variability. During a major depressive episode, functional limitations are prevalent, affecting over 90% of individuals with major depressive disorder (MDD) in at least one domain [28-31]. These limitations manifest in various aspects of daily life, including work functioning, as any individuals with MDD struggle to maintain productivity, concentration, and decision-making abilities at work, which may result in absenteeism, reduced work quality, or difficulty meeting job requirements; Social interactions, as MDD often leads to social withdrawal, reduced communication, and challenges in forming or maintaining relationships. Individuals may avoid social gatherings, isolate themselves, or experience conflicts in interpersonal relationships; Self-care activities and basic self-care tasks such as grooming, hygiene, and nutrition can become challenging. Individuals may neglect personal care routines due to a lack of motivation, energy, or interest. Managing household responsibilities (i.e., chores) may become overwhelming. Tasks such as cleaning, cooking, and organizing may be neglected, leading to an untidy or disorganized living environment; Recreational activities, such as interest in hobbies, leisure activities, and enjoyable pursuits often diminishes. Individuals may lose motivation to engage in activities they once found pleasurable, resulting in decreased participation in recreational pursuits. Students or employees with MDD may experience difficulties in academic or work performance; Concentration problems, memory deficits, and decreased motivation may lead to academic underachievement or work-related challenges. MDD disrupts daily routines, resulting in irregular sleep patterns, disrupted eating habits, and overall disorganization. Individuals may struggle to maintain consistent routines and may experience fluctuations in energy levels throughout the day. The severity of symptoms in MDD accounts for a limited percentage of the variability in functional impairment, indicating that other factors contribute to the impact on daily functioning [32]. Additionally, studies collectively highlight the need for a holistic understanding of MDD, considering its impact on both occupational functioning and cognitive dimensions [33]. 

Cognitive deficits and challenges in everyday functioning are observed in various severe mental disorders, and managing mood symptoms is crucial for better functional outcomes. This aligns with the process as described by [54] of mental mechanisms, a chain of processing information. Therefore, cognitive impairment is considered an independent dimension in various psychiatric disorders, highlighting its importance as a potential target for treatment. Deficiencies in cognitive performance and the inability to function in everyday life are observed in MDD and other severe mental disorders. Enhancing the management of mood symptoms is crucial for better functional outcomes, but cognitive deficits also exert a substantial, partially independent impact on psychosocial functioning. Similarly, a critical review from 2015 mentions that cognitive symptoms come from cognitive functions, which makes them the main characteristics of depressive disorders [55]. Moreover, notable improvements in daily occupations are observed during remission; however, achieving remission in MDD may not guarantee genuine recovery, emphasizing the importance of focusing on functioning as a key measure of treatment success [40-41]. Factors beyond remission, including the combination of pharmacotherapy with psychotherapy, can directly impact functioning, highlighting the need for a comprehensive approach to treatment. Psychotherapy should be considered for an increased focus on functioning as a vital measure of treatment success, putting emphasis on evaluating the effectiveness of treatment in assisting patients with their daily occupational activities. 

Researchers have observed contradictory findings challenging the interconnection between functional disability patterns and depressive symptoms, emphasizing that mood symptoms alone may not entirely explain the extent of functional impairment. Also, mood symptoms alone may not completely account for the extent of functional impairment. Furthermore, evidence and perspectives suggest that there is variation in functional disability patterns based on the severity of depression [42-43]. This underscores the importance of assessing adaptive functioning as an integral part of treating depression to enhance intervention effectiveness, especially for individuals seeking a successful return to their pre-illness lifestyle. Parallel, a study in 2020 concluded that there is a further need for an improvement in communication between patients with MDD and healthcare providers to set appropriate treatment goals and promote symptomatic and functional recovery, which supports the findings of this review [56]. Additionally, there is an increasing emphasis on functional recovery as a target in clinical treatment for MDD, advocating for accurate measurement and quantification of adaptive functioning [44]. However, improvements in functionality may not consistently align with enhancements in depressive symptoms [43]. Less than 5% of clinical trial research for MDD has employed scales to assess functional levels, indicating a notable gap in the understanding and evaluation of functional aspects of depression [31].

Psychotherapy, defined by the American Psychiatric Association, aims to modify behaviors, cognitions, and emotions for individuals with MDD. MDD is recognized globally as a leading psychiatric condition, causing work-related disability and disruptions in daily functioning [45]. Despite limited research on the impact of psychotherapy on occupational improvements in MDD [20], studies show its effectiveness in enhancing overall functioning and quality of life [46-47]. This is shown by addressing work-related stresses and psychological effects such as depression. While combined treatment is considered more effective, both psychotherapy and pharmacotherapy alone demonstrate efficacy; it improves depressive symptoms, overall functioning, social interactions, work productivity, and engagement in daily activities [47].

Evidence indicates that individuals who prefer psychological therapy may choose not to receive any treatment rather than opt for medication [47]. Furthermore, recent studies indicate supportive evidence stating that combined treatment is the most effective option [57]. However, their research is based on quantitative data that measures symptom relief instead of supporting the wellbeing and overall quality of life of the patient. All in all, research emphasizes the need for tailored therapies to address the specific needs of individuals with MDD.

Based on the findings of the literature review, future research directions may include longitudinal studies to examine the long-term effects of psychotherapy on occupational functioning in individuals with MDD. Additionally, comparative effectiveness research could further elucidate which psychotherapeutic approaches are most efficacious in improving specific aspects of daily occupations. Furthermore, studies exploring the integration of technology-based interventions and workplace accommodations for individuals with MDD may provide valuable insights into innovative treatment strategies.

Overall, the study sheds light on the multifaceted impact of MDD on daily occupations and the potential of psychotherapy to enhance occupational functioning in affected individuals. By addressing these issues comprehensively, clinicians and researchers can contribute to improving the quality of life and functional outcomes for individuals living with MDD.

## Conclusions

People with MDD exhibited impairments in various adaptive functions, including communication, daily living skills, and socialization. Hence, MDD significantly impairs an individual's capacity to engage in daily activities and maintain their usual baseline level of functioning. This aligns with numerous research that indicate that MDD negatively impacts multiple adaptive functions related to family, school, interpersonal relationships, and general health, leading to a decline in overall quality of life. While current treatment strategies primarily target the reduction or elimination of MDD symptoms, this study review highlights the limitations of a symptom-focused approach and advocates for an expanded focus on enhancing functioning to promote overall well-being in MDD patients. Contrary to common expectations, this review highlights that improvements in functioning did not continue beyond the acute treatment phase, and there is even a notable decline after one year of follow-up. This underscores the importance of integrating measures of functioning into both research and clinical efforts for MDD, aiming to enhance long-term functioning alongside symptom alleviation. Therefore, successful MDD psychological treatment necessitates research into and implementation of specific and personalized interventions designed to improve and restore functioning, complementing evidence-based MDD psychological treatments.
